# About Birds

**Published:** 1887-08-20

**Authors:** F. O. Morris

**Affiliations:** Rector of Nunburnholme, Yorkshire; author of "A History of British Birds," etc


### Incidents of Animal Life.

Collated by the Rev. F. O. MORRIS, B.A.,
Rector of Nunburnholme, Yorkshire; author of "A History of British Birds," etc.

"He prayeth best, who loveth best
All things both great and small;
For the dear God who loveth us,
He made and loveth all."—Coleridge.

The following anecdotes are extracted from letters which I have received at various times, and they cannot, I think, fail to prove of interest to many readers of The Hospital.

### About Birds.

Some time since you referred, I believe, to the skylark as having become comparatively a *rara avis* in your locality. I was congratulating myself about the same time on an unusual number having arrived here. When I was appointed to the charge of this parish, about seven years ago, I missed nothing so much in my walks through the fields as the bright, cheery song of the lark, as he would be ever springing up at one's feet, and mounting into the sky, in my old parish; but last spring, however, I met with many, although I did not think their voice so strong or striking as in the Eastern counties.

It is sad to see so many of these merry little songsters strung up in bundles at the shops of the game-sellers, and put to so untimely an end for the sake of so small a morsel!

With reference to swallows, a pair took a fancy to a window-curtain in my bedroom for their nest. The material—a kind of chintz—not being sufficiently sharp to hold their clay, the latter came down again and again when it became dry. How long the little fellows would have continued to labour in vain I cannot say, but their perseverance led me to see if I could not help them. So I drew some threads through the curtain where they had been at work, and let them hang loosely down about an inch or two; the next day these were wrought into their work, and their nest was finished within a few hours. Shortly afterwards an egg was in the nest, and when two or three had been laid the hen bird would sit on its edge as if to guard its precious contents during the night. At early dawn her mate would come and "twit" at the window to call her out for breakfast. This state of things lasted until the number of eggs was laid, and she began to sit. Then her mate took up his quarters in the room during the night on the top of my bedstead, and it was most amusing to see and hear him, as soon as daylight began to dawn, warbling his little song of praise with all the graceful gesticulation of the most eloquent orator. This done, he would fly to the window and want to be let out. For his convenience the window would be left open—about four inches space—and through this he would soon be seen returning with some breakfast for his mate—sweeping in at almost railway speed, and without touching anything until he reached the nest. And then what a chattering there would be! They reared their young family without any mishap, and left us. Whether it was the same pair that came to the same room, and reared another family the following spring, I cannot tell, but from their having felt so much at home, I used to think it was the same.

If I am not tiring you too much, I have something to tell you about a fieldfare, or as the peasants call it, a "dow-felfer," which has been affording me some amusement of late. In front of my study window there is a hawthorn full of berries. This tree the bird took possession of during the early autumn—for, I presume, its winter's supply of food when none was to be had elsewhere—and the watchfulness with which he guards this tree is something marvellous. Although they used to make their nests every year in my orchard in Suffolk, I have not seen these birds here until this season. No bird can trespass on this tree without the first possessor swooping down upon it. Robins, sparrows, and hedge-sparrows alight on it sometimes without any evil intention, but only to be driven out as soon as there. A pair of blackbirds, which have long made this garden their home, sometimes try to steal a berry or two from the forbidden tree, when I have seen the old bird coming from the further end of the park at express speed, and driving them out in a twinkling. Birds may hop about with impunity at about eight or ten yards from the tree, but no nearer, or the fieldfare is down upon them like a thunderbolt. Why this particular tree should have become such a bone of contention I cannot conceive, as there are plenty of berries elsewhere near at hand. Is it because it is in front of our windows, where it can see (for the sake of companionship or fancied protection) or be seen? I have been informed that a white swallow has been frequently seen in the valley of the Wharfe this year.

---

We live just outside the small town of Harrow, and have a garden, of about four acres only, with a high wooden paling. We have left purposely for birds part of it uncultivated, namely, a thick hedge, two ponds, and a few trees; on these we have placed boxes with lids on them for tits' nests, in which they build regularly and get very tame. Last year we had twenty-seven nests in the garden alone; this year very nearly as many, and three fresh kinds of birds who have not built here before. A marsh-tit has a nest in a hole it made in an old stump of a tree, and the bird hisses and pecks the hand of anyone who comes near the hole. We have for the first time a red-backed shrike's nest, which allows us to watch her while sitting and observe her large prominent eye. We have a blackcap, also for the first time. We have two blue-tits in the boxes with ten eggs in each; they allow us to take the boxes off the tree and carry them about to be looked at. We have, besides, a great-tit in a box, a robin in a box, and a willow-wren just hatched. A robin has just hatched in a place under a bend of a pipe close to the front door where people pass constantly, and she lets us feed her and the young birds, though occasionally she flies at and pecks people who go too near, in the boldest way. We keep several foreign ducks, some of which build, but we find it very difficult to rear them. We had three nests of wild ducks, all hatched, and a Carolina duck also reared some successfully a few years ago. I noticed larks in March soaring, but not since. We found in a wood in the neighbourhood a blackcap's nest, with five eggs of her own and a cuckoo's egg, of which she was so proud that she would only hop off close to the nest while we looked at it. Another year we found a cuckoo's egg in a garden-warbler's nest which was bold enough to peck our hands when near the nest. We found a nightingale's nest near there too, and generally we find three or four in that locality. One of our boys on the training ship for the navy, at Dartmouth, has found no less than three buzzard's nests this spring. My husband once helped a nut-hatch in a battle to secure a hole for a nest which a starling incessantly occupied, by turning out the starling's nest till the nut-hatch succeeded in plastering up too much clay for the starling to pass through. Another time a brown owl attacked him on the back as he was going up to see the young birds, and hit him quite hard. The same bird, later on, he found wounded in a tree, surrounded by a larder of birds and mice brought there to support her by her mate, as she had been unable to fly for some time.

(*To be continued.*)

